# Association between gut microbiota and onset of type 2 diabetes mellitus: a two-sample Mendelian randomization study

**DOI:** 10.3389/fcimb.2024.1327032

**Published:** 2024-03-26

**Authors:** Hongyan Zhang, Li Ma, Wenbo Peng, Bing Wang, Yongning Sun

**Affiliations:** ^1^ Department of Traditional Chinese Medicine, Shanghai Sixth People’s Hospital Affiliated to Shanghai Jiao Tong University School of Medicine, Shanghai, China; ^2^ Shaanxi Key Laboratory of Research on Traditional Chinese Medicine Physical Constitution and Diseases Prevention and Treatment, Shaanxi University of Chinese Medicine, Xianyang, Shaanxi, China; ^3^ Department of Endocrinology, Shaanxi Provincial Hospital of Traditional Chinese Medicine, Xi’an, Shaanxi, China; ^4^ Department of Cardiology, Shanghai Municipal Hospital of Traditional Chinese Medicine, Shanghai University of Traditional Chinese Medicine, Shanghai, China

**Keywords:** gut microbiota, type 2 diabetes mellitus, Mendelian randomization, causal association, species

## Abstract

**Aim:**

Mendelian randomization (MR) analysis has been used in the exploration of the role of gut microbiota (GM) in type 2 diabetes mellitus (T2DM); however, it was limited to the genus level. This study herein aims to investigate the relationship of GM, especially at the species level, with T2DM in order to provide some evidence for further exploration of more specific GM taxa and pathway abundance in T2DM.

**Methods:**

This two-sample MR study was based on the summary statistics of GM from the available genome-wide association study (GWAS) meta-analysis conducted by the MiBioGen consortium as well as the Dutch Microbiome Project (DMP), whereas the summary statistics of T2DM were obtained from the FinnGen consortium released data. Inverse variance weighted (IVW), MR-Egger, strength test (*F*), and weighted median methods were used to examine the causal association between GM and the onset of T2DM. Cochran’s *Q* statistics was employed to quantify the heterogeneity of instrumental variables. Bonferroni’s correction was conducted to correct the bias of multiple testing. We also performed reverse causality analysis.

**Results:**

The corrected IVW estimates suggested the increased relative abundance of family *Oxalobacteraceae* (OR = 1.0704) and genus *Oxalobacter* (OR = 1.0874), respectively, were associated with higher odds of T2DM, while that of species *faecis* (OR = 0.9460) had a negative relationship with T2DM. The relationships of class *Betaproteobacteria*, family *Lactobacillaceae*, species *finegoldii*, and species *longum* with T2DM were also significant according to the IVW results (all *P* < 0.05).

**Conclusions:**

GM had a potential causal association with T2DM, especially species *faecis*, *finegoldii*, and *longum*. Further studies are still needed to clarify certain results that are contradictory with previous findings.

## Introduction

Type 2 diabetes mellitus (T2DM) is growing at an alarming speed globally in the 21st century (International Diabetes Federation (IDF), 2022). T2DM as well as its complications have brought a heavy burden of disease in all regions (Ali et al., 2022). Identifying the factors that have a causal relationship with the development of T2DM can provide an important evidence base for disease prevention and facilitate the development of new treatment strategies.

The gut microbiota (GM) is a complex ecosystem and consists of approximately 4 × 10^13^ species of symbiotic bacteria, protozoa, fungi, archaea, and viruses (Chen et al., 2021; Martino et al., 2022). GM is involved in a variety of physiological activities in the human body, such as metabolism, inflammatory processes, and immune responses (Fan and Pedersen, 2021; Gill et al., 2022). Increasing evidence have shown that GM plays an important role in metabolic diseases such as T2DM (Gurung et al., 2020). Patients with T2DM have metabolic disorders and chronic inflammatory states accompanied by disturbances in the GM (Yang et al., 2021). A significant association of changes in the composition profile of GM with the development of T2DM as well as related complications have also been found (Iatcu et al., 2021)—for example, the disequilibrium of phylum *Bacteroidetes/Firmicutes* has been associated with increased intestinal permeability, with bacteria byproducts infiltrating through a leaky gut barrier triggering subsequent inflammatory responses characteristic of DM (Iatcu et al., 2021). Several bacteria, such as *Lactobacillus fermentum*, *plantarum* and *casei*, *Roseburia intestinalis*, *Akkermansia muciniphila* and *Bacteroides fragilis*, have also been reported to exert a protective role *via* reducing the risk of DM development through decreasing proinflammatory markers and maintaining intestinal barrier integrity (Iatcu et al., 2021). Nevertheless, it is necessary to distinguish between the characteristics of the GM that cause the disease and those caused by the disease or its treatment.

Mendelian randomization (MR) is a valuable tool to assess the causality of an observed relationship between a modifiable exposure or risk factor and a clinically relevant outcome (Sekula et al., 2016). Due to Mendelian law of segregation and independent classification, it can eliminate confounding bias in comparison to traditional observational epidemiological studies and facilitate the separation of causal pathways for phenotypic grouping risk variables that are difficult to randomize or susceptible to measurement errors (Davies et al., 2018). Moreover, MR results are less susceptible to bias caused by reverse causation because the genetic code is not influenced by environmental factors or preclinical diseases (Skrivankova et al., 2021).

In recent years, MR analysis has been gradually applied to explore the causal association of GM with the risk of T2DM (Sanna et al., 2019; Yuan et al., 2023). Current studies have been limited to exploring the causal association between the abundance of GM in specific families or genera and the occurrence of T2DM. Yang et al. (2018) identified genus *Acidaminococcus*, *Aggregatibacter*, *Anaerostipes*, *Blautia*, *Desulfovibrio*, *Dorea*, and *Faecalibacterium* to be nominally linked to T2DM. Xiang et al. (2022) suggested that *Streptococcaceae* was associated with a higher risk of T2DM in European populations, whereas there was a causal relationship between *Acidaminococcaceae* and T2DM in Asian populations. Most previous cohorts relied on 16s ribosomal RNA (rRNA) measurements, which could not allow bacterial or pathway abundance identification at the species level. In fact, the measurement of species and pathway abundance is essential for further investigation on the GM in an individual because even being placed in the same genus does not guarantee metabolic consistency in the physiological process (Vieira-Silva et al., 2016; Lopera-Maya et al., 2022).

This study herein performed a two‐sample MR analysis to investigate the potential causal relationship of GM with the occurrence of T2DM based on all existing GM taxa (including phylum, class, order, family, genus, and species) in order to provide some evidence-based evidence on T2DM prevention.

## Methods

### Data sources

**Figure 1:**
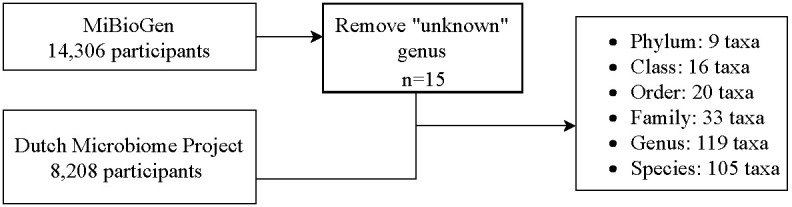
Flowchart of the study procedure.

In this two-sample MR study, data on genome-wide association studies (GWASs) were extracted for GM and T2DM. **Figure 1** shows a flowchart of the research procedure. GM taxa including phylum, class, order, family, and genus levels were extracted from the MiBioGen consortium (Kurilshikov et al., 2021), whereas the species-level data were extracted from the Dutch Microbiome Project (DMP) (Lopera-Maya et al., 2022). Data on patients with T2DM were extracted from the FinnGen consortium (Yuan and Larsson, 2022). More details on the source of exposures and outcomes are shown in **Table 1**.

**Table 1:** Information of the data source for GM and T2DM.

Variables	Consortium	Trait	Year	Population	Sample size	SNPs	Taxa	Websites
Exposures	MiBioGen	Phylum	2021	European	14,306		9	https://mibiogen.gcc.rug.nl/menu/main/home
		Class	2021	European	14,306		16	
		Order	2021	European	14,306		20	
		Family	2021	European	14,306		33	
		Genus	2021	European	14,306		119	
	DMP	Species	2021	European	7,738		105	https://dutchmicrobiomeproject.molgeniscloud.org/menu/main/home
Outcome	Finngen	T2DM	2021	European	215,654	16,380,440		https://www.finngen.fi/en

GM, gut microbiota; T2DM, type 2 diabetes mellitus; SNP, single-nucleotide polymorphisms; DMP, Dutch Microbiome Project.

This study was conducted in accordance with the local legislation and institutional requirements. The participants have provided their written informed consent to participate in each GWAS. The requirement of ethical approval was waived by the Shanghai Municipal Hospital of Traditional Chinese Medicine for the studies involving humans because these databases used in our study were publicly available, and all data were de-identified.

### Single-nucleotide polymorphism selection

We first selected single-nucleotide polymorphisms (SNPs) that significantly associated with GM as potential instrument variables (IVs). The threshold to select IVs was *P* < 1.0 × 10^-6^. We then removed SNPs with minor allele frequency (MAF) ≤0.01. The linkage disequilibrium (LD) threshold was set to be *r* ^2^ = 0.001, with a clumping distance of 10,000 kb. The MR-Egger regression test was used to monitor potential horizontal pleiotropy effect, namely, the confounding effect resulted from other diseases, which may violate the second assumption in MR analysis. The intercept item of MR-Egger that was significant represents the existence of pleiotropy. Additionally, palindromic SNPs were deleted according to the principle of MR to ensure that the same allele corresponds the effects between SNPs and the exposure and that on the outcome.

### The assumptions of MR analysis

MR must conform to three important assumptions to minimize the impact of bias on the results. First, IVs must be independent of confounders related to both exposure and outcome. Second, IVs should be significantly associated with the exposure. We estimated the association strength between GM and IVs according to the formula: F = ((N – K - 1)/K) * (r^2/^(1 - r^2^)), r^2^ = 2 * EAF * (1 - EAF) * β^2^/SD^2^, where *β* was the regression coefficient for GM and IVs, EAF was the effect allele frequency, *K* was the number of IVs, *N* was the sample size, and SD was the standard difference. A weak association between IVs and exposure is recognized when *F <*10. Third, IVs influence outcomes through exposure only, that is, no horizontal pleiotropy effect of IVs on outcome.

### Statistical analysis

The statistical analyses were performed by using R version 4.2.0 (Institute for Statistics and Mathematics, Vienna, Austria). The R package “TwoSampleMR” was used to explore the potential causal association between GM and T2DM. The statistical significance of evidence for the potential causal effect was indicated by *P* < 0.05. In the calculation for the causal effect values, inverse variance weighted (IVW) test was used, which is the primary method to obtain unbiased estimates when horizontal pleiotropy was absent. The evaluation indexes were odds ratios (ORs) and 95% confidence intervals (CIs). Cochrane’s *Q* test was used to test heterogeneity. IVs with *P <*0.05 were recognized as heterogeneous. When the associations were still significant by Bonferroni’s correction method, that is corrected *P* < 0.05/*n*, where *n* was the number of taxa in different levels, indicating that the potential causal relationship was reliable. The weighted-median method was utilized to provide a robust and consistent estimate of the effect, even if nearly 50% of the genetic variants were invalid instruments. The intercept of MR-Egger regression examined the presence of potential pleiotropy in IVs (*P* > 0.05 was deemed to have no horizontal pleiotropy). We used robust adjusted profile score (MR-RAPS) analysis to produce reliable inferences about systemic and specific pleiotropy when weak instruments were present.

## Results

**Figure 2:**
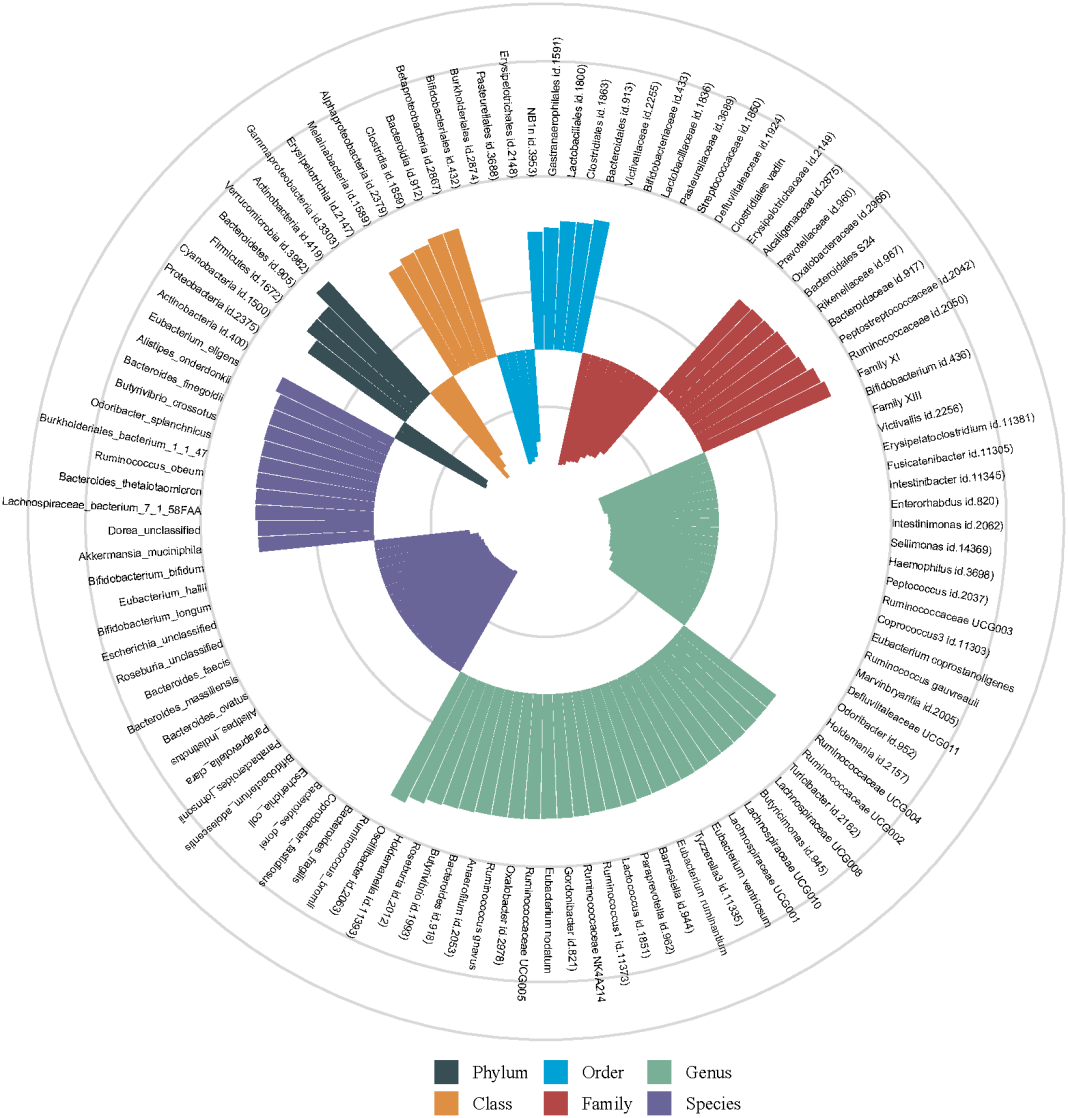
Potential causal relationships of gut microbiota (GM) abundances with the risk of type 2 diabetes mellitus (T2DM). The different colored histograms represent the different levels of GMs. The outward orientation of the column represents the GM as a potential risk factor for T2DM (OR > 1), whereas the inward orientation represents that as a potential protective factor (OR < 1).

**Figure 3:**
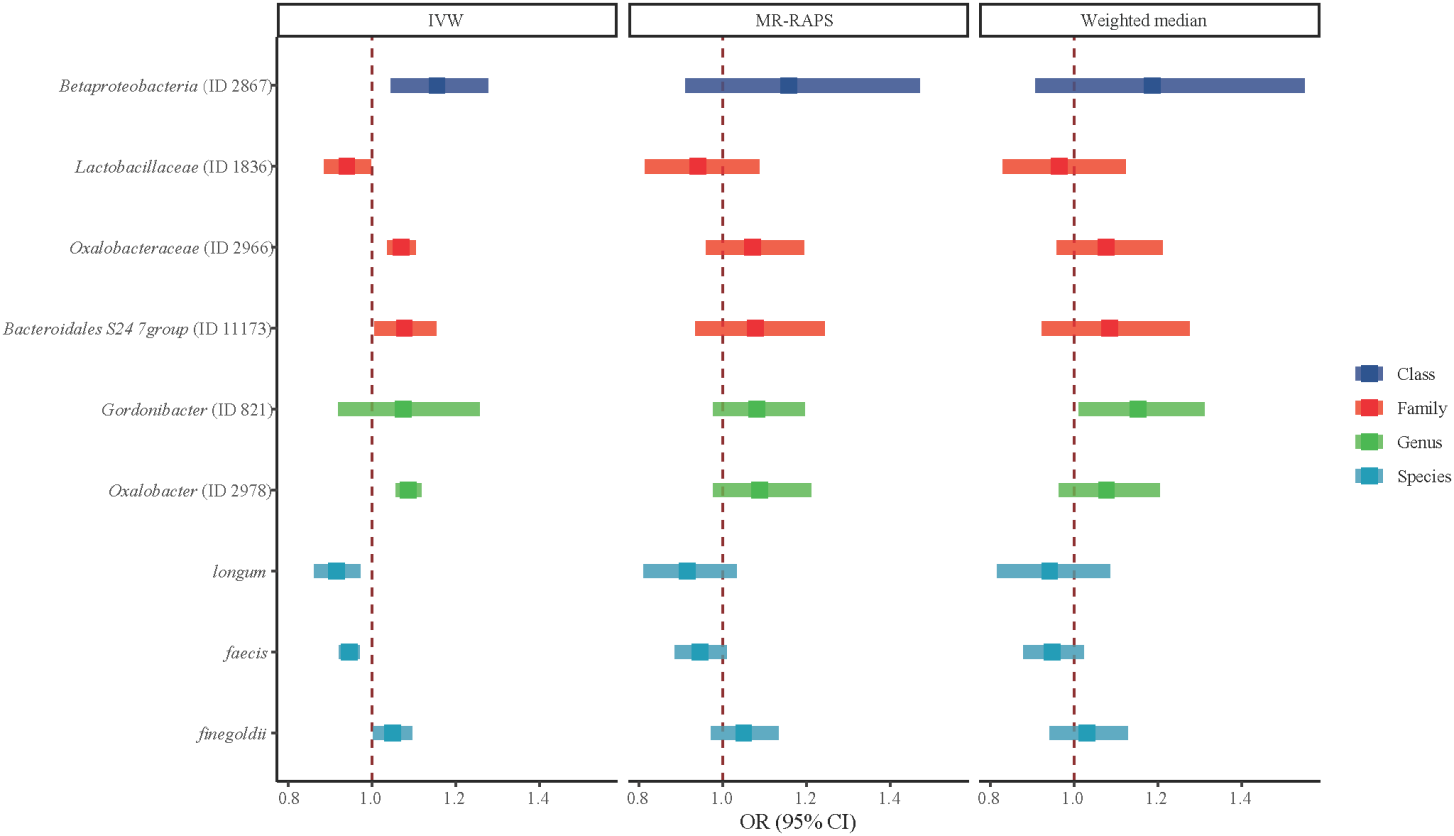
Potential causal associations between gut microbiota (GM) in different levels and type 2 diabetes mellitus (T2DM). Potential associations of class, family, genus, and species levels of GM with T2DM assessed using inverse variance weighted, MR-RAPS, and weighted median methods. The dark blue color represents class level, the red color represents family level, the green color represents genus level, and the light blue color represents species level.

**Figure 2** shows the association of GM abundance at different levels with the risk of T2DM. Basing on the circular chart, we found that there were opposite relationships between different GM taxa and T2DM inside the same level. **Table 2** similarly shows the potential causal relationships between GM at different levels and T2DM. To be specific, the increased relative abundances of family *Oxalobacteraceae* (OR = 1.0704) and genus *Oxalobacter* (OR = 1.0874) were both significantly associated with higher odds of T2DM, whereas that of species *faecis* had a negative relationship with T2DM (OR = 0.9460). Moreover, the IVW test suggested that the increased relative abundances of class *Betaproteobacteria* (OR = 1.1560) and species *finegoldii* (OR = 1.0493) were linked to higher odds of T2DM, while those of family *Lactobacillaceae* (OR = 0.9405) and species *longum* (OR = 0.9158) were associated with lower odds of T2DM. Similarly, **Figure 3** clearly reflects the potential causal association from GM at different levels to the odds of T2DM occurrence.

**Table 2:** Association between GM and the risk of T2DM.

GM levels	Trait	Test method	SNPs	OR (95% CI)	*P*
Significant					
Family	*Oxalobacteraceae* (ID 2966)	IVW	4	1.0704 (1.0365–1.1053)	3.33*10^-5^
		MR-Egger		0.9258 (0.3332–2.5720)	0.8960
		MR-RAPS		1.0705 (0.9593–1.1946)	0.2232
		Weighted median		1.0771 (0.9595–1.2091)	0.2079
Genus	*Oxalobacter* (ID 2978)	IVW	4	1.0874 (1.0577–1.1180)	2.98*10^-9^
		MR-Egger		1.2638 (0.6424–2.4863)	0.5676
		MR-RAPS		1.0876 (0.9775–1.2102)	0.1230
		Weighted median		1.0777 (0.9593–1.2106)	0.2077
Species	*faecis*	IVW	3	0.9460 (0.9217–0.9709)	2.79*10^-5^
		MR-Egger		0.8906 (0.7223–1.0981)	0.4744
		MR-RAPS		0.9458 (0.8857–1.0099)	0.0957
		Weighted median		0.9484 (0.8792–1.0232)	0.1713
Suggestive significant					
Class	*Betaproteobacteria (ID 2867)*	IVW	3	1.1560 (1.0460–1.2777)	0.0045
		MR-Egger		0.8522 (0.2539–2.8602)	0.8388
		MR-RAPS		1.1574 (0.9114–1.4698)	0.2306
		Weighted median		1.1864 (0.9154–1.5378)	0.1965
Family	*Bacteroidales S24 7group (ID 11173)*	IVW	3	1.0777 (1.0068–1.1536)	0.0312
		MR-Egger		1.2226 (0.7986–1.8719)	0.5248
		MR-RAPS		1.0781 (0.9353–1.2427)	0.2995
		Weighted median		1.0855 (0.9254–1.2733)	0.3134
Family	*Lactobacillaceae (ID 1836)*	IVW	3	0.9405 (0.8858–0.9987)	0.0452
		MR-Egger		0.9805 (0.6481–1.4833)	0.9408
		MR-RAPS		0.9402 (0.8137–1.0865)	0.4033
		Weighted median		0.9652 (0.8215–1.1340)	0.6665
Genus	*Gordonibacter (ID 821)*	IVW	3	1.0759 (0.9206–1.2574)	0.3575
		MR-Egger		1.3818 (0.8015–2.3824)	0.4519
		MR-RAPS		1.0810 (0.9771–1.1958)	0.1307
		Weighted median		1.1524 (1.0093–1.3157)	0.0360
Species	*finegoldii*	IVW	3	1.0493 (1.0038–1.0969)	0.0333
		MR-Egger		1.3046 (0.7568–2.2490)	0.5140
		MR-RAPS		1.0499 (0.9723–1.1336)	0.2140
		Weighted median		1.0313 (0.9399–1.1315)	0.5150
Species	*longum*	IVW	3	0.9158 (0.8627–0.9723)	0.0039
		MR-Egger		0.7519 (0.4105–1.3775)	0.5255
		MR-RAPS		0.9153 (0.8114–1.0326)	0.1502
		Weighted median		0.9419 (0.8169–1.0862)	0.4106

Significant represents test by Bonferroni’s correction (P < 0.05/n). Suggestive significant represents test by IVW test (P < 0.05).

GM, gut microbiota; T2DM, type 2 diabetes mellitus; SNP, single-nucleotide polymorphism; OR, odds ratio; CI, confidence interval; IVW, inverse variance weighted test.

**Table 3** shows the results of the pleiotropy and heterogeneity tests. We confirmed the impact of relatively accurate MR results, that is, the potential causal relationship of GM with T2DM by the sensitivity analyses. No horizontal pleiotropy and heterogeneity were observed in the potential causal associations between the relative abundances of family *Oxalobacteraceae*, genus *Oxalobacter*, and species *faecis* and the odds of T2DM (MR-Egger *P >*0.05 and Cochrane’s *Q* test *P >*0.05). Besides this, no horizontal pleiotropy and heterogeneity existed in the potential causal relationships between class *Betaproteobacteria*, species *finegoldii*, family *Lactobacillaceae*, and species *longum* and T2DM (MR-Egger *P >*0.05, and Cochrane’s *Q* test *P >*0.05).

**Table 3:** Hypothesis testing of GM taxa significantly associated with T2DM.

GM levels	Trait	Strength		Pleiotropy test		Heterogeneity test			
		*F* value	*r* ^2^ (%)	Egger intercept	*P*	Q Egger	*P*	Q IVW	*P*
Significant									
Family	*Oxalobacteraceae* (ID 2966)	26.58	0.7	0.0175	0.8059	0.2139	0.8986	0.2922	0.9615
Genus	*Oxalobacter* (ID 2978)	27.76	0.8	-0.0199	0.7027	0.0367	0.9818	0.2305	0.9725
Species	*Faecis*	27.76	0.1	0.015	0.6607	0.0137	0.9067	0.3618	0.8345
Suggestive significant									
Class	*Betaproteobacteria* (ID 2867)	24.39	0.5	0.0245	0.7038	0.1543	0.6945	0.4063	0.8161
Family	*Bacteroidales S24 7group* (ID 11173)	26.31	0.6	-0.0138	0.6506	0.1449	0.7035	0.5188	0.7715
Family	*Lactobacillaceae* (ID 1836)	24.86	0.5	-0.0048	0.8692	0.3495	0.5544	0.3929	0.8216
Genus	*Gordonibacter* (ID 821)	26.15	0.5	-0.0384	0.5189	2.7319	0.0984	5.1573	0.0759
Species	*Finegoldii*	25.31	0.1	-0.0427	0.5741	0.1398	0.7085	0.7648	0.6822
Species	*Longum*	26.50	0.1	0.0237	0.6332	0.1412	0.7071	0.5632	0.7546

Significant represents test by Bonferroni’s correction (P < 0.05/n). Suggestive significant represents test by IVW test (P < 0.05).

GM, gut microbiota; T2DM, type 2 diabetes mellitus; F = ((N – K - 1)/K) * (r^2^/(1 - r^2^)), r^2 = ^2 * EAF * (1 - EAF) * β^2^/SD^2^.

In addition, **Table 4** shows the results of the reverse causality analysis. The relative abundance of family *Lactobacillaceae* had a negative reverse causal association with high odds of T2DM (MR-Egger OR = 0.8236, *P* = 0.0141); however, no reverse causality has been found between different GM taxa and T2DM.

**Table 4:** Reverse causality between GM and the risk of T2DM.

GM levels	Trait	Test method	OR (95% CI)	*P*
Significant				
Family	*Oxalobacteraceae* (ID 2966)	IVW	1.0287 (0.9611–1.1012)	0.4143
		MR-Egger	1.0702 (0.8983–1.2750)	0.4529
		MR-RAPS	1.0307 (0.9614–1.1049)	0.3944
		Weighted median	1.0348 (0.9064–1.1813)	0.6128
Genus	*Oxalobacter* (ID 2978)	IVW	1.0287 (0.9520–1.1115)	0.4744
		MR-Egger	1.1290 (0.9388–1.3577)	0.2057
		MR-RAPS	1.0226 (0.9509–1.0996)	0.5472
		Weighted median	1.0471 (0.9194–1.1926)	0.4878
Species	*Faecis*	IVW	1.0023 (0.8846–1.1357)	0.9709
		MR-Egger	0.7618 (0.5482–1.0587)	0.1168
		MR-RAPS	1.0034 (0.8638–1.1657)	0.9643
		Weighted median	0.9062 (0.7222–1.1371)	0.3951
**Suggestive significant**			1.0046 (0.9718–1.0385)	0.7863
Class	*Betaproteobacteria* (ID 2867)	IVW	1.0048 (0.9156–1.1027)	0.9202
		MR-Egger	1.0090 (0.9702–1.0494)	0.6542
		MR-RAPS	1.0437 (0.9748–1.1174)	0.2198
		Weighted median	1.0046 (0.9718–1.0385)	0.7863
Family	*Bacteroidales S24 7group* (ID 11173)	IVW	0.9823 (0.9298–1.0377)	0.5227
		MR-Egger	0.9679 (0.8417–1.1131)	0.6500
		MR-RAPS	0.9646 (0.9129–1.0192)	0.1995
		Weighted median	0.9984 (0.9050–1.1014)	0.9739
Family	*Lactobacillaceae* (ID 1836)	IVW	1.0354 (0.9658–1.1100)	0.3268
		MR-Egger	0.8236 (0.7104–0.9548)	0.0141
		MR-RAPS	1.0259 (0.9680–1.0873)	0.3884
		Weighted median	0.9511 (0.8621–1.0494)	0.3179
Genus	*Gordonibacter* (ID 821)	IVW	1.0097 (0.9303–1.0959)	0.8173
		MR-Egger	0.9964 (0.8132–1.2209)	0.9727
		MR-RAPS	0.9968 (0.9166–1.0840)	0.9398
		Weighted median	1.0204 (0.8821–1.1805)	0.7856
Species	*Finegoldii*	IVW	0.9907 (0.8663–1.1330)	0.8916
		MR-Egger	0.8321 (0.6203–1.1161)	0.2290
		MR-RAPS	0.9776 (0.8620–1.1087)	0.7245
		Weighted median	0.8715 (0.7067–1.0747)	0.1983
Species	*Longum*	IVW	0.9500 (0.8909–1.0130)	0.1174
		MR-Egger	0.9123 (0.7804–1.0664)	0.2576
		MR-RAPS	0.9750 (0.9131–1.0412)	0.4500
		Weighted median	0.9399 (0.8421–1.0491)	0.2691

Significant represents test by Bonferroni’s correction (P < 0.05/n). Suggestive significant represents test by IVW test (P < 0.05).

GM, gut microbiota; T2DM, type 2 diabetes mellitus; OR, odds ratio; CI, confidence interval; IVW, inverse variance weighted test.

## Discussion

We conducted a two-sample MR analysis to investigate the potential causal relationship of GM with the occurrence of T2DM. The study results showed that the relative abundances of class *Betaproteobacteria*, family *Oxalobacteraceae*, genus *Oxalobacter*, species *faecis*, species *finegoldii*, and species *longum* had potential causal associations with the odds of T2DM.

In recent years, there was only a limited number of research focused on the relationship of GM with T2DM on the basis of MR method. Yang et al. (2018) used separate-sample MR to obtain estimates of the associations of 27 genera of GM with T2DM and other metabolic diseases, which identified *Acidaminococcus*, *Aggregatibacter*, *Anaerostipes*, *Blautia*, *Desulfovibrio*, *Dorea*, and *Faecalibacterium* as being nominally associated with T2DM. Xiang et al. (2022) conducted MR analysis to investigate whether GM (in family level) was causally linked to T2DM risk and found that an increased relative abundance of *Streptococcaceae* was associated with a higher risk of T2DM in the European population. Recently, Sun et al. (2023) performed a two-sample MR study to explore the causal relationship of GM with T2DM, demonstrating that genus *Alistipes*, genus *Allisonella*, genus *Flavonifractor*, and genus *Haemophilus* acted as defense elements against T2DM, whereas family *Clostridiaceae1*, family *Coriobacteriaceae*, genus *Actinomyces*, and genus *Candidatus Soleaferrea* were risk factors for T2DM. In clinical practice, since even being placed in the same genus does not guarantee metabolic consistency in the physiological process, the measurement of species and pathway abundance is essential for further investigation on the GM in individuals. Compared with Sun’s research, although we similarly investigated the causal association of GM with T2DM, we further explored these relationships in the species level of GMs and found that the increased relative abundance of *finegoldii* was associated with higher odds of T2DM (OR = 1.0493). The GM features significantly associated with odds of T2DM which we observed were different and less than those in Sun’s study, which was possibly due to the fact that we set the threshold of IVs selection to *P* < 1.0 × 10^-6^ that was stricter. In addition, Bonferroni’s correction method was used to correct the causal relationships of GM with T2DM in our study, which can further correct the bias of multiple testing and made our findings more robust. Herein the difference in genus and species level may be beneficial to the development of microbial agents related to the treatment and prevention of T2DM—for example, specific species of GM can be very helpful in developing ideas on customized or personalized medicine (Popoviciu et al., 2023). However, another MR research on the impact of GM and associated metabolites on cardiometabolic traits, chronic diseases, and human longevity showed that their results cannot support a large causal impact of GM features on T2DM (Gagnon et al., 2023). In conclusion, our results could only indicate that there may be potential causal associations of GM at different levels with T2DM, and further basal and prospective cohort studies are needed to reveal the real roles of GM in the occurence of T2DM in the future.

Specifically, we observed that the increased relative abundance of class *Betaproteobacteria*, family *Oxalobacteraceae*, and genus *Oxalobacter* were all associated with higher odds of T2DM, whereas that of family *Lactobacillaceae* had a negative causal association with T2DM. The role of *Betaproteobacteria* in T2DM has not been reported (Larsen et al., 2010; Camargo et al., 2020). However, *Blautia* and *Desulfovibrio* in Yang’s study (Yang et al., 2018) and *Haemophilus* in Sun’s study (Sun et al., 2023) were potential risk factors for T2DM, which all belong to phylum *Proteobacteria*. A previous cross-sectional study in Japanese adults identified the *Blautia* genus as a commensal bacterium that is inversely correlated with T2DM, and a possible underlying mechanism was that its amino acid metabolites conferred anti-adipogenesis and anti-inflammatory properties to adipocytes (Hosomi et al., 2022). Another study in obese patients with T2DM found that the relative abundance of *Roseburia* species was increased after surgery among those achieving diabetes remission (Murphy et al., 2017). Furthermore, *Haemophilus* may affect the occurrence and development of T2DM *via* involving the body’s inflammatory response (Brueggemann et al., 2021; Lopez-Lopez et al., 2021). In an animal experiment of whole grain, fermentation affects the GM composition of T2DM; the researchers found that the abundance of family *Oxalobacteraceae* was increased, whereas genus *Lactobacillus* was decreased in mice fed a high-fat, high-fructose diet (the T2DM model), which is consistent with our findings (Costabile et al., 2022). Metabolic dysfunction was linked to proportionally higher levels of *Proteobacteria* (especially *Oxalobacteraceae*) and decreased *Lactobacilli* (Nguyen et al., 2015; Do et al., 2018). Nevertheless, the specific mechanisms of these bacteria taking part in T2DM progress are not clear so far, and the population-based studies are still lacking such that we cannot make reliable speculations due to these species differences.

Regarding the species level, *finegoldii* belongs to the genus *Alistipes*, which may be pathogenic (Zhao et al., 2020b). We found that the increased abundance of *finegoldii* was associated with higher odds of T2DM; however, Sun’s study came to the opposite conclusion, that is, the genus *Alistipes* acted as a defense element against T2DM. According to the animal experiment by He et al. (2022), the probiotic-mediated anti-obesity effect was considered associated with members of *Alistipes finegoldii*. Another population-based study also showed a negative association between the glycemic parameter and the abundance of *Alistipes finegoldii* (Companys et al., 2022). The underlying mechanism of the potential causal relationship between *Alistipes finegoldii* and T2DM has not been clarified. A possible reason for the opposite results between the current study and those in previous studies may be the pathogenesis of T2DM itself. *Alistipes* has protective effects against various diseases, such as liver fibrosis, cancer immunotherapy, colitis, and cardiovascular disease, but, in contrast, it is pathogenic in colorectal cancer and depression (Parker et al., 2020). In addition, we also found that the increased abundance of *faecis* and *longum* was negatively associated with high odds of T2DM. The role of *faecis*, a type of bacteria from human feces, in the pathogenesis of T2DM is still unknown. Among patients with chronic diseases, such as chronic kidney disease (Lohia et al., 2022), Chrohn’s disease (Bao et al., 2022), and gastrointestinal cancers (Li et al., 2021), the abundance of *Roseburia faecis* is obeserved to be significantly reduced. The more consistent speculation about the mechanism by which *Roseburia faecis* play a beneficial role in these diseases is that it can ferment dietary fiber into butyrate, which was considered to be a protector of the gut (Wang et al., 2012). The species *longum* was also significantly decreased in patients with both T1DM and T2DM, which was associated with the G protein-coupled receptor (GPR) 43 and GPR41 gene expression (Demirci et al., 2022). *longum* may also lower the levels of fasting blood glucose as well as alleviate insulin resistance in diabetic mice, enhancing the anti-oxidative capacity, increasing the hepatic glycogen content, decreasing the gene expression levels of glucose-6-phosphatase (G6Pase) and phosphoenolpyruvate carboxykinase (PEPCK) in the livers, and thus regulating the disturbance of GM (Hao et al., 2022). Moreover, *longum* has been made as a variety of pharmaceutical and probiotic supplements in recent years (Zhao et al., 2020a; Gou et al., 2023).

MR is a relatively superior study design to clarify the causal effect of potential risk factors on diseases of interest. By investigating the potential causal association of GM with the occurrence of T2DM, our study may provide some rederences for further exploration on flora regulation methods that benefit prevention and treatment in T2DM in clinical practice, which could effectively reduce the incidence and social burdens of T2DM. Compared to previous MR studies, the current study is more comprehensive because we first included the species level of GM in the analyses. However, there are still some limitations in this study. Our study is limited in the European population, so the causal association between GM and T2DM in other races needs to be further explored. GWAS on GM is still in the initial stage, so that the sample size, as well as the number of SNPs, is relatively small. Due to the small sample size and insufficient efficacy of the microbiome GWAS, there may not be enough IVs for certain bacterial characteristics at the genus or species level. Certain results may be contradicting due to age, dietary patterns, lifestyle behaviors, ethnicity, and geographical location because GM is influenced by multiple factors (Nitzan et al., 2023). However, we could not obtain information on the characteristics of the subjects because it was not available in the MIMIC-IV database.

## Conclusion

There was a potential causal association between the relative abundance of GM and the risk of T2DM. The relative abundances of class *Betaproteobacteria*, family *Oxalobacteraceae*, genus *Oxalobacter*, and species *finegoldii* had positive associations with the occurrence of T2DM, whereas those of species *faecis* and *longum* had negative ones. However, certain results that are contradictory with previous findings needed further clarification.

## Data availability statement

Publicly available datasets were analyzed in this study. This data can be found here: The GWAS Catalog, https://www.ebi.ac.uk/gwas/.

## Ethics statement

The requirement of ethical approval was waived by Shanghai Municipal Hospital of Traditional Chinese Medicine for the studies involving humans. The studies were conducted in accordance with the local legislation and institutional requirements. The participants provided their written informed consent to participate in this study.

## Author contributions

HZ: Conceptualization, Funding acquisition, Project administration, Supervision, Writing – original draft, Writing – review & editing. LM: Data curation, Formal analysis, Funding acquisition, Investigation, Methodology, Writing – review & editing. WP: Data curation, Formal analysis, Investigation, Methodology, Writing – review & editing. BW: Data curation, Formal analysis, Investigation, Methodology, Writing – review & editing. YS: Conceptualization, Supervision, Writing – review & editing.
